# Secular trends in incidence of invasive beta-hemolytic streptococci and efficacy of adjunctive therapy in Quebec, Canada, 1996-2016

**DOI:** 10.1371/journal.pone.0206289

**Published:** 2018-10-23

**Authors:** Antoine Couture-Cossette, Alex Carignan, Adam Mercier, Claudine Desruisseaux, Louis Valiquette, Jacques Pépin

**Affiliations:** 1 Department of Microbiology and Infectious Diseases, Université de Sherbrooke, Sherbrooke, Quebec, Canada; 2 Hôpital Maisonneuve-Rosemont, Montréal, Quebec, Canada; 3 Hôpital Charles-Lemoyne, Longueuil, Quebec, Canada; Hospital Universitari de Bellvitge, SPAIN

## Abstract

**Objectives:**

To examine secular changes in the incidence of invasive beta-hemolytic streptococcal infections, and to assess the efficacy of immunoglobulins and clindamycin as adjunctive therapies in the management of *Streptococcus pyogenes* infections.

**Methods:**

Retrospective cohort study of all cases of invasive group A (GAS), B (GBS), C or G (GCGS) streptococcal infections managed in a Canadian tertiary center from 1996–2016. Population incidence was measured for diabetics and non-diabetics. Adjusted odds ratios (AOR) and their 95% confidence intervals (CI) were calculated by logistic regression.

**Results:**

741 cases were identified (GAS: 249; GBS: 304; GCGS: 188). While the incidence of invasive GAS infections fluctuated with no clear trend, incidence of invasive GBS and GCGS increased over time and were 8.4 and 6.3 times higher in diabetics. Mortality of invasive GAS infections decreased from 16% (6/37) in 1996–2001 to 4% (4/97) in 2011–15. Among patients with GAS infections, clindamycin administered concomitantly with a beta-lactam within 24 hours of admission decreased mortality (AOR: 0.04, 95%CI: 0.003–0.55, *P* = 0.02. Immunoglobulins had no such effect (AOR: 1.66, 95%CI: 0.16–17.36, *P* = 0.67). The protective effect of clindamycin was similar in patients with pneumonia/empyema compared to all others.

**Conclusion:**

Incidence of GBS and GCGS infections increased due to an expansion of the high-risk population (elderly diabetics), but also rose in non-diabetics. No such secular change was seen for invasive GAS infections. The decrease in mortality in patients with invasive GAS infections presumably reflects better case-management. Adjunctive clindamycin reduced mortality in invasive GAS infections; immunoglobulins did not, but power was limited. The highest mortality was seen in patients with GAS pneumonia/empyema, for whom clindamycin was protective but underused.

## Introduction

While several reports have documented an increase in the incidence of invasive non-group A β-hemolytic streptococcal infections, the underlying reasons remain ill-defined [1–5]. In our center, we noted an increase of invasive group G streptococcal (iGGS) infections, which prompted this study aimed at comparing the incidence, severity and mortality of invasive β-hemolytic streptococcal infections over a 20-year period. Also, while adding clindamycin to a beta-lactam is recommended for invasive group A β-hemolytic streptococcal (iGAS) infections based on in vitro data and animal models, the clinical benefits of this adjuvant therapy are scarcely documented [6–7]. Furthermore, the efficacy of adjuvant intravenous immunoglobulin (IVIG) administration remains debated as there is variation between lots in their capacity to neutralize streptococcal superantigens and no established proof of their benefit [8–10]. This study aimed to assess the efficacy of immunoglobulins and clindamycin as adjunctive therapies in the management of *Streptococcus pyogenes* infections., but also to examine secular changes in the incidence of invasive beta-hemolytic streptococcal infections.

## Methods

### Design and setting of the study

Sherbrooke is the main city of the Estrie region in the province of Quebec, Canada. The populations of Sherbrooke and Estrie were respectively 164,666 and 322,099 inhabitants in 2015. The Centre Hospitalier Universitaire de Sherbrooke (CHUS) is a university center encompassing two hospitals (Hôtel-Dieu and Fleurimont) totaling 712 beds. As the region’s only tertiary care center, five community hospitals located in Estrie refer cases to CHUS, which also gets transfers from outside the region. In a single-payer public healthcare system, these characteristics ensure that virtually all severe acute illnesses within its catchment area are referred to CHUS.

This retrospective cohort study aimed to identify all cases of invasive infections due to beta-hemolytic streptococci diagnosed and managed at CHUS between January 1^st^, 1996 and June 30^th^, 2016. Approval to review hospital records and to access the regional database of reportable diseases was granted by the CHUS ethics committee. Potential cases were identified by examining all microbiology results reporting the presence of β-hemolytic streptococci. Specimens from throat, urine, eye, ear, sinus or superficial wounds were excluded. All isolates obtained from blood, cerebrospinal, pleural or synovial fluids were deemed to represent an invasive infection, excluding aspirates from bursitis cases. For lower respiratory tract isolates, cases with radiographic evidence of pneumonia with no other respiratory pathogen recovered were considered as having invasive streptococcal pneumonia. Similarly, isolates from deep surgical specimens collected from sterile sites were retained only if recovered in pure growth, or along with a non-pathogenic commensal species. Surgical site infections were excluded unless a necrotizing infection was recognized. We considered as significant the presence of group A (GAS), C (GCS) or G (GGS) streptococci in genital specimens from cases with endometritis or puerperal sepsis as the main discharge diagnosis (group B streptococci [GBS], a frequent vaginal colonizer, was excluded). For iGAS, cases with incomplete microbiologic data were identified by cross-referencing the provincial reportable diseases database.

Laboratory identification of streptococci was performed as followed: all isolates displaying large colony size (>0.5 mm in diameter after 24 hours), positive catalase test, and β-hemolysis on 5% sheep blood agar were further tested for serogroup specificity using a rapid agglutination test (Prolex Strep Grouping Kit, Pro-Lab, Canada).

### Definitions

Past medical history was collected to calculate the Charlson score [11], along with demographic, microbiological, clinical and therapeutic data. Severe sepsis was defined as hypotension with end-organ dysfunction responding to volume repletion, while patients not responding to volume expansion were considered as having septic shock. An increase in serum creatinine of ≥50% from the baseline defined acute renal failure. The presence of acute pulmonary edema was based on radiography while acute respiratory distress syndrome was considered present in patients meeting the Berlin definition [12]. Streptococcal toxic shock syndrome (STSS) was defined according to standard criteria [13]. Clindamycin and immunoglobulin exposure was considered in patients who received at least one dose of treatment. The outcome used for multivariate analysis was in-hospital infection-related mortality, defined as cases for whom death was a direct consequence of streptococcal infection or one of its immediate complications and happened within the index hospitalization.

### Data analysis

Calculations of incidence rates were limited to a subpopulation for which we were confident of catching all cases: residents of Sherbrooke and adjacent towns (population in 2015: 197,867) and from 2002 onwards, at which time both hospital sites merged their electronic records. Other analyses included all identified cases. Regional data on the prevalence of diabetes were obtained from the Institut National de Santé Publique du Québec which tabulates such statistics from physicians billing claims [14].

Incidence rates were calculated using population denominator data from the Institut de la Statistique du Québec (Provincial statistics institute), and age-standardized according to the 1991 Canadian standard population [15–17]. Simple linear regression and correlation coefficients were employed to examine the association between incidence rates and time periods. Univariate and multivariate Poisson regression analyses were conducted to estimate incidence rate ratios of invasive beta-hemolytic streptococcal infections for different periods of observation, as well as for adults with or without diabetes.

Data were analysed with Stata 12.0 (Stata Corporation, College Station, Texas). Proportions were compared with the χ^2^ or Fisher’s test, as appropriate. Non-normally distributed continuous variables were compared using the Kruskal-Wallis test. Crude and adjusted odds ratios (AOR) and their 95% confidence intervals (CI) were calculated by logistic regression. Multivariable analyses were performed starting with the variable most strongly associated with the outcome, and sequentially adding variables until no other variable reached significance. Variables where at least one stratum was significantly (*P*≤0.05) different from baseline as per the Wald test were retained. As substantial confounding was expected between several variables, each one of those examined in univariate analyses was tested in the multivariate model regardless of its statistical significance in univariate analyses. The AOR presented in the multivariate models are those calculated after adjustment for all variables retained in this specific model. Since there were too many categories for anatomical sites of infection, these were grouped into: fasciitis/myositis, pneumonia/empyema, and all others. ICU admission was used as an overall measure of disease severity to allow for a proper measurement of the effect of therapeutic modalities (clindamycin, immunoglobulins) preferentially used in the very sick patients, including those that did not satisfy criteria for STSS.

## Results

### Incidence and descriptive epidemiology

A total of 741 invasive β-hemolytic streptococcal infections were identified: 249 corresponded to GAS, 304 to GBS, 48 to GCS and 140 to GGS. Groups C and G, mostly representing the same subspecies (*Streptococcus dysgalactiae* ssp *equisimilis*) except for rare, generally zoonotic isolates, will be addressed as GCGS. Seventy-seven cases were referred from outside the region, while 664 occurred in Estrie (of which 502 came from the subpopulation [Sherbrooke and adjacent towns] used to calculate incidence rates). Overall, 84% of cases had a positive blood culture.

Table 1 presents the annual incidence rates per 100,000 inhabitants of all invasive GAS, GBS and GCGS infections for each two-year period, as well as over the whole (2002–2015) period of observation. The incidence rates of bacteremic infections are also displayed.

**Table 1:** Incidence rates per 100,000 inhabitants per year for invasive and bacteremic streptococcal infections.

	Incidence of invasive infections			Incidence of bacteremia		
	Group A Streptococcus	Group B Streptococcus	Group C or G Streptococcus	Group A Streptococcus	Group B Streptococcus	Group C or G Streptococcus
2002–03	5.6	3.6	1.9	3.1	3.1	1.9
2004–05	3.8	3.8	2.7	3.3	5.5	2.7
2006–07	5.7	4.3	3.8	3.5	3.8	3.5
2008–09	5.0	3.2	5.0	3.5	2.9	4.2
2010–11	3.1	8.6	4.1	2.1	7.0	3.9
2012–13	4.5	6.6	5.6	3.3	5.8	4.8
2014–15	7.2	9.7	6.7	6.2	8.7	6.2
2002–15	5.0	6.0	4.3	3.6	5.3	3.9

Over the fourteen years (2002–2015), the incidence rates of iGAS infections were respectively 4.4, 4.4 and 8.0 per 100,000 in the 0–17, 18–64 and ≥65 years age groups. For iGBS infections, the incidence rates were respectively 2.9, 3.6 and 19.0 per 100,000 in the same age groups while for iGCGS infections these rates were 0.2, 2.1 and 18.1 per 100,000.

**Fig 1:**
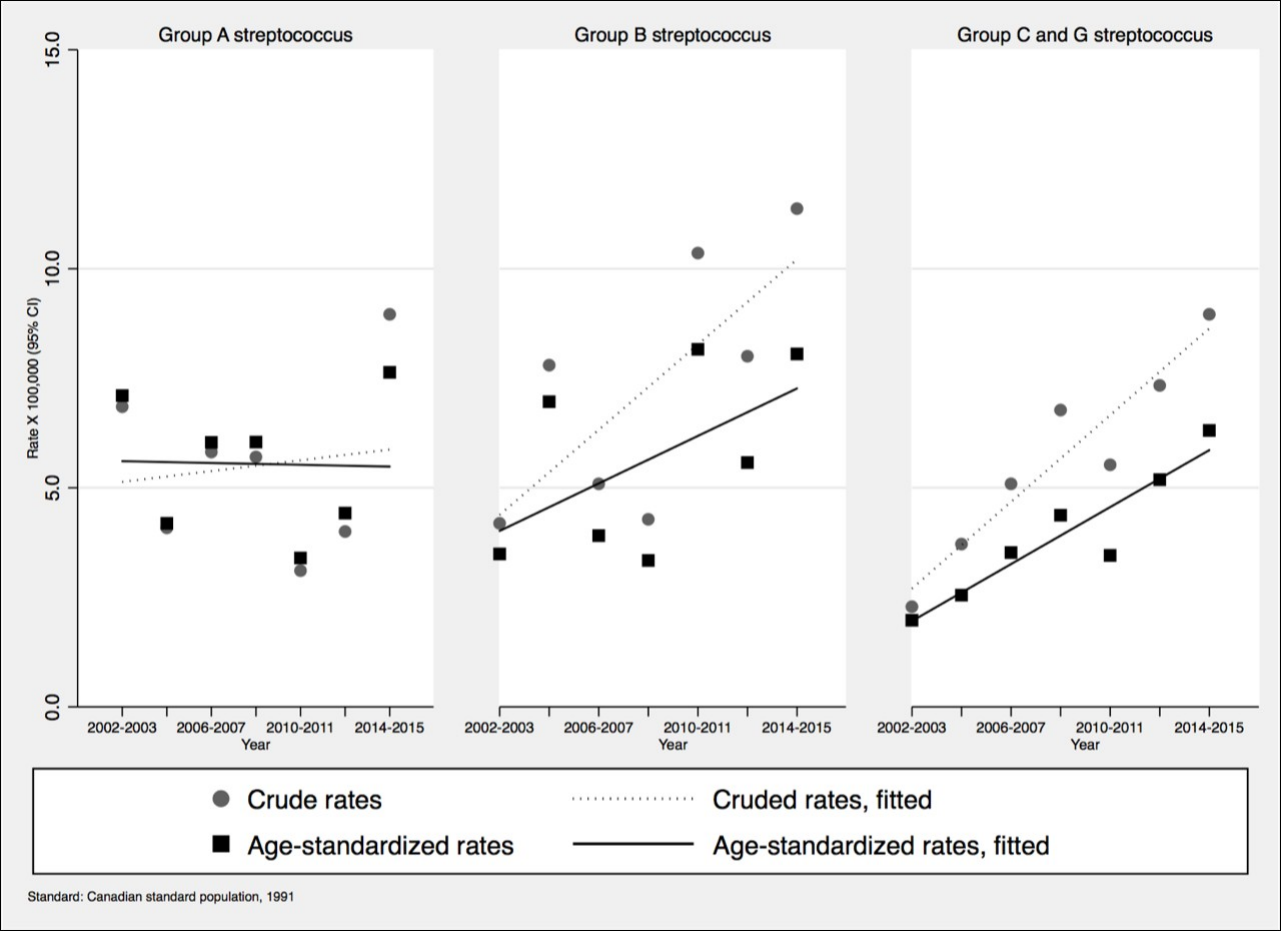
**Incidence of invasive infections per 100,000 inhabitants, Sherbrooke and adjacent towns, 2002–2015 (Fig 1A: group A *Streptococcus*; 1B: group B *Streptococcus*; 1C: groups C and G *Streptococcus*).** Crude and age standardized rates are displayed.

**Fig 2:**
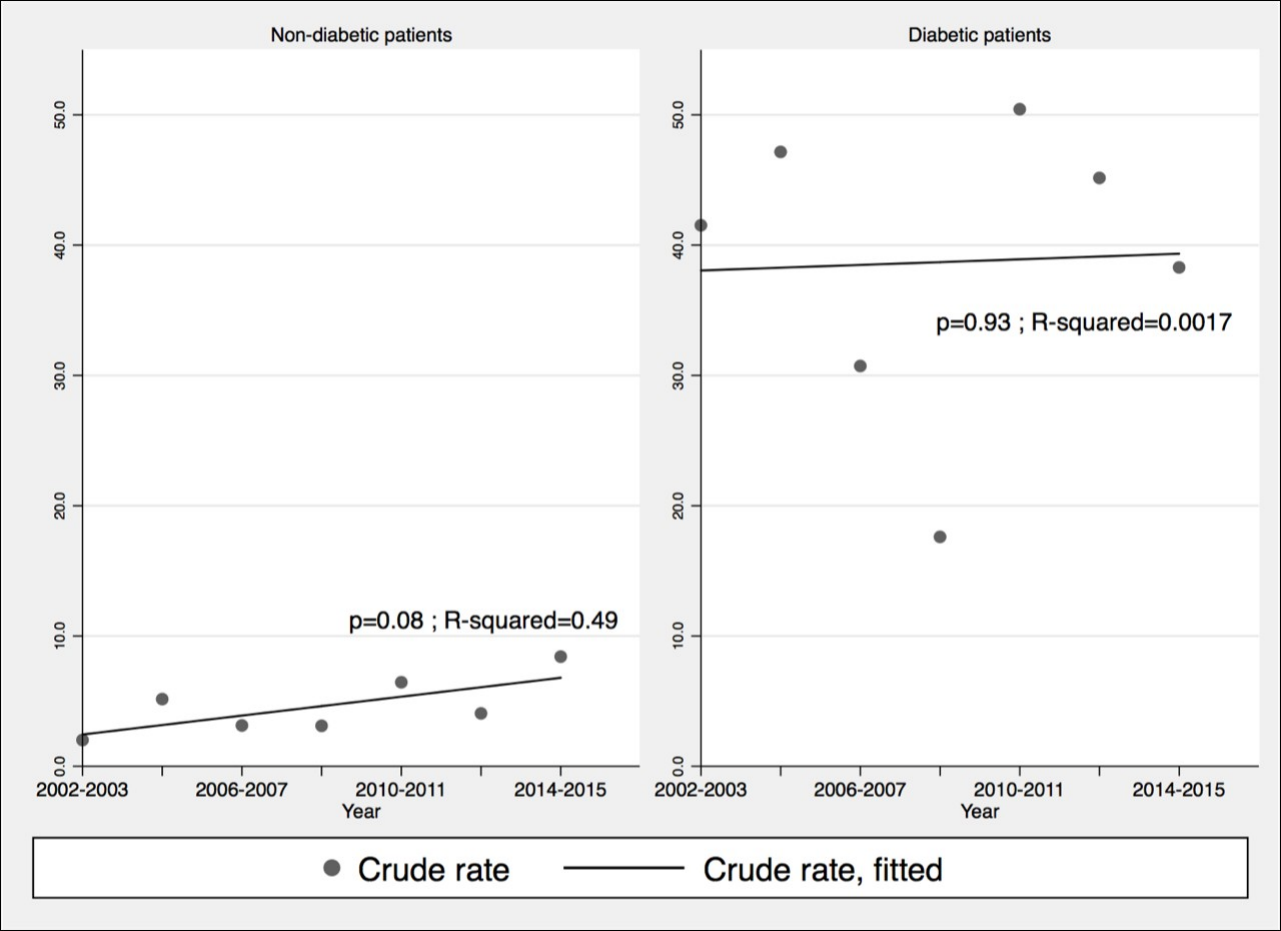
Incidence of invasive group B *Streptococcus* infections per 100,000 inhabitants, according to the presence or absence of diabetes, Sherbrooke and adjacent towns, 2002–2015. Crude rates are displayed.

**Fig 3:**
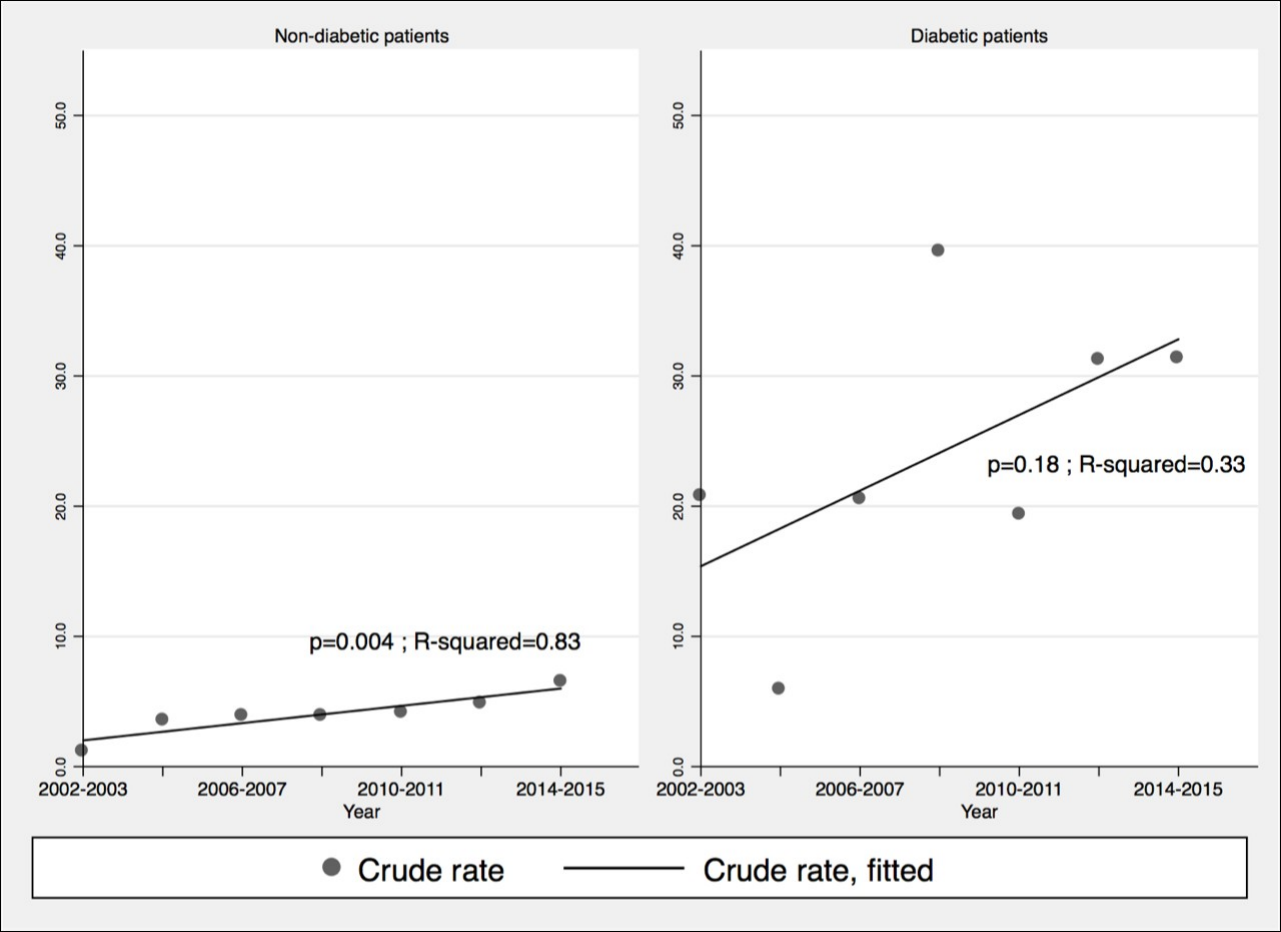
Incidence of invasive groups C and G *Streptococcus* infections per 100,000 inhabitants, according to the presence or absence of diabetes, Sherbrooke and adjacent towns, 2002–2015. Crude rates are displayed.

The incidence of iGAS infections fluctuated with no clear trend (Fig 1) (p = 0.95, R-squared = 0.0008). Incidence of iGCGS and iGBS infections increased over time, significantly only for the former (p = 0.0002; R-squared = 0.87, versus for GBS p = 0.2, R-squared = 0.31) but this was less marked after standardizing for age, implying that some of the increase was due to aging of the population. The incidence of iGBS and iGCGS infections was much higher in diabetics than in non-diabetics. Throughout the 2002–2015 period of observation, the incidence rates of iGAS, iGBS and iGCGS infections were respectively 1.6 (95%CI: 0.88–2.82; *P* = 0.5), 8.4 (95%CI: 6.02–11.65; *P* 0.001) and 6.3 (95%CI 4.30–9.29; *P*<0.001) times higher in diabetics than non-diabetics. After adjusting for diabetes prevalence, the increased incidence of iGBS (*P* < .001) and iGCGS infections (*P* < .001) remained significant. The incidence of iGBS and iGCGS increased among non-diabetic individuals but not in their diabetic counterparts (Figs 2 and 3).

### Clinical presentation

Table 2 summarizes the clinical characteristics by Lancefield group organism. While iGAS infections spanned all age groups, very few children were infected with other groups, apart from cases of neonatal GBS sepsis. Consequently, there were considerable differences in the median age of patients and the burden of co-morbidities summarized within the Charlson score. Cases of iGCGS predominated among males. As expected, clindamycin, intravenous immunoglobulins and surgical interventions were used mainly in patients with iGAS infections. The prevalence of comorbidities was compared between groups. After stratifying for age, few differences were seen (data not shown) apart from diabetes mellitus. Among patients aged 40–65 years, the prevalence of diabetes was 8% (5/61) among cases of GAS, 42% (41/98) for GBS and 42% (21/50) for GCGS (p<0.001). Among those aged ≥65 years, diabetes was present in 31% (19/62) of patients infected with GAS, 41% (52/128) for GBS and 34% (41/121) for GCGS (*P* = 0.05).

**Table 2:** Clinical characteristics of patients with invasive streptococcal infections.

	Group A Streptococcus (n = 249)	Group B Streptococcus (n = 304)	Groups C or G Streptococcus (n = 188)	p-value^a^
**Age group (years)**				
<1	3 (1.2%)	52 (17.1%)	0	
1–7	47 (18.9%)	1 (0.3%)	1 (0.5%)	
18–64	137 (55.0%)	123 (40.5%)	66 (35.1%)	
65–79	33 (13.3%)	77 (25.3%)	63 (33.5%)	
≥80	29 (11.6%)	51 (16.8%)	58 (30.9%)	
**Median (IQR)**	42.7 (23.7–64.9)	59.7 (38.3–74.6)	70.7 (61.9–82.0)	<0.001
**Sex**				
Female	134 (53.8%)	141 (46.4%)	72 (38.3%)	
Male	115 (46.2%)	163 (53.6%)	116 (61.7%)	
**Charlson score**				
0	146 (58.6%)	99 (32.6%)	40 (21.3%)	
1–2	52 (20.9%)	83 (27.3%)	41 (21.8%)	
3–5	32 (12.9%)	72 (23.7%)	64 (34.0%)	
≥6	19 (7.6%)	50 (16.4%)	43 (22.9%)	
**Median (IQR)**	0 (0–2)	2 (0–4.5)	3 (1–5)	<0.001
**Clinical diagnosis**				
Cellulitis	51 (20.5%)	67 (22.0%)	97 (51.6%)	
Fasciitis/myositis	35 (14.1%)	3 (1.0%)	4 (2.1%)	
Bone & Joint	24 (9.6%)	36 (11.8%)	23 (12.2%)	
Genital tract or GI	35 (14.1%)	60 (19.7%)	18 (9.6%)	
Central nervous system	4 (1.6%)	20 (6.6%)	0	
Pneumonia/empyema	62 (24.9%)	36 (11.8%)	16 (8.5%)	
Endovascular	12 (4.8%)	25 (8.2%)	13 (6.9%)	
Primary bacteremia	12 (4.8%)	53 (17.4%)	17 (9.0%)	
Other	14 (5.6%)	4 (1.3%)	0	
**Streptococcal**				
**toxic shock syndrome**	54 (21.7%)	11 (3.6%)	2 (1.1%)	<0.001
**Adjunctive treatments**				
Clindamycin	121 (48.6%)	23 (7.6%)	23 (12.2%)	<0.001
Immunoglobulins	35 (14.1%)	4 (1.3%)	3 (1.6%)	<0.001
**Surgical intervention**^b^				
None	153 (61.4%)	242 (79.6%)	155 (82.4%)	<0.001
Major	57 (22.9%)	0	7 (3.7%)	
Minor	39 (15.7%)	62 (20.4%)	26 (13.8%)	

IQR: inter-quartile range; GI: gastro-intestinal

^a^ p-value comparing the distribution of characteristics between groups A, B and CG

^b^ Interventions defined as “major” were those considered as life-, limb- or organ-sparing (*e*.*g*. fasciotomy, drainage of empyema, hysterectomy), as opposed to ‘minor’ (*e*.*g*. joint lavage, abscess drainage for patients not meeting toxic shock criteria).

Table 3 shows the frequency of complications. Although some complications were more frequent with GAS, there was no difference in the infection-related mortality or in the duration of hospitalization.

**Table 3:** Clinical course of patients with invasive streptococcal infections.

	Group A Streptococcus (n = 249)	Group B Streptococcus (n = 304)	Groups C or G Streptococcus (n = 188)	p-value
Severe sepsis on admission	57 (22.9%)	60 (19.7%)	57 (30.3%)	0.04
Septic shock on admission	67 (26.9%)	47 (15.5%)	26 (13.8%)	<0.001
Intensive care unit admission	123 (49.4%)	100 (32.9%)	44 (23.4%)	<0.001
Median (IQR) length of hospital stay, days	9 (5–16)	10 (5–20)	11 (8–18.5)	NS
**Complications**				
Acute renal failure	78 (31.3%)	97 (31.9%)	78 (41.5%)	0.005
Vasopressors administered^a^	57 (22.9%)	43 (14.1%)	23 (12.2%)	0.004
Intubation	65 (26.1%)	45 (14.8%)	16 (8.5%)	<0.001
Pulmonary edema	38 (15.3%)	35 (11.5%)	24 (12.8%)	NS
ARDS	23 (9.2%)	14 (4.6%)	2 (1.1%)	<0.001
**Sequelae**				
Chronic renal failure	3 (1.2%)	2 (0.7%)	7 (3.7%)	0.03
Amputation	2 (0.8%)	9 (3.0%)	4 (2.1%)	NS
Organ resection	5 (2.0%)	1 (0.3%)	0	NS
Neurological	9 (3.6%)	17 (5.6%)	5 (2.7%)	NS
Tracheostomy	2 (0.8%)	4 (1.3%)	5 (2.7%)	NS
**Death related to infection**	20 (8.0%)	24 (7.9%)	17 (9.0%)	NS
**Relapse/Reinfection**^b^				
Relapse	3 (1.2%)	6 (2.0%)	4 (2.1%)	NS
Reinfection (same group)	0	10 (3.3%)	4 (2.1%)	0.02
Reinfection (other group)	2 (0.8%)	7 (2.3%)	4 (2.1%)	NS

IQR: inter-quartile range; ARDS: acute respiratory distress syndrome; NS: not significant

^a^ Some patients with septic shock did not receive vasopressors, either because they had a terminal illness and no aggressive therapy was initiated, or because they died very quickly before vasopressors could be started

^b^ Relapse: a second episode caused by the same pathogen within 3 months of the previous one; reinfection: a second episode more than 3 months after the previous one

### Analysis of risk factors for mortality and estimation of treatment effect

Twenty patients died from iGAS disease, 10 of whom within 48 hours of hospital admission (8 who did not receive clindamycin, two who did), while the longest interval was 32 days. Table 4 summarizes the case-fatality ratio among patients with iGAS infection according to various characteristics. As expected, mortality increased with age and Charlson score. Mortality decreased over time, with the lowest rate seen in the latest period (from 2011 to 2016). It was highest among patients with a pneumonia and/or empyema (9/62, 14.5%). Mortality was lower among patients given clindamycin (combined with a beta-lactam in 94% of cases, and for a median duration of 5 days) within 24 hours of admission. No such effect on mortality was seen in patients given immunoglobulins (odds ratio: 1.09, 95% CI: 0.30–3.92, p = 1.00). These therapeutic modalities were used preferentially in sicker patients. Among patients admitted to the ICU, 69% (85/123) received clindamycin, 26% (32/123) were given immunoglobulins and 48% (59/123) underwent surgery, while among those treated on a ward these percentages were 29% (37/126) for clindamycin, 2% (3/126) for immunoglobulins and 32% (40/126) for surgery. While the use of clindamycin did not change over time (48–49% for each five-year period), immunoglobulins lost favour: 9/37 (24%) patients received it in 1996–2000, 12/55 (22%) in 2001–2005, 8/60 (13%) in 2006–2010 and 6/97 (6%) in 2011–2016. However, the use of critical care increased: 14/37 (38%) in 1996–2000, 26/55 (47%) in 2001–2005, 29/60 (48%) in 2006–2010 and 54/97 (56%) in 2011–2016. Clindamycin administration and its timing varied substantially according to the anatomical site of infection (Table 5).

**Table 4:** In-hospital infection-related mortality in patients with invasive group A streptococcal infections according to various characteristics.

	Deaths/Total (%)	Crude odds ratio (95% CI)	p-value	Adjusted odds ratio (95% CI)	p-value
**Year of diagnosis**					
1996–2000	6/37 (16.2)	1.00		1.00	
2001–2005	3/55 (5.5)	0.30 (0.07–1.28)	NS	0.02 (0.002–0.35)	0.006
2006–2010	7/60 (11.7)	0.68 (0.21–2.21)	NS	0.26 (0.04–1.59)	NS
2011–2016	4/97 (4.1)	0.22 (0.06-.84)	0.03	0.01 (0.001–0.15)	0.001
**Age (years)**					
		1.06 (1.03–1.09)^a^	<0.001	1.11 (1.06–1.16)^a^	<0.001
0–17	0/50 (0.0)				
18–64	5/137 (3.6)				
65–79	6/33 (18.2)				
≥80	9/29 (31)				
**Sex**					
Female	12/134 (9.0)	1.00			
Male	8/115 (7.0)	0.76 (0.30–1.93)	NS		
**Charlson score**					
0	7/146 (4.8)	1.24 (1.06–1.44)^b^	0.008		
1–2	4/52 (7.7)				
3–5	6/32 (18.8)				
≥6	3/19 (15.8)				
**Site of infection**					
All others	9/152 (5.9)	1.00		1.00	
Fasciitis/myositis	2/35 (5.7)	0.96 (0.20–4.67)	NS	2.08 (0.15–29.09)	NS
Pneumonia/empyema	9/62 (14.5)	2.70 (1.01–7.16)	0.05	4.41 (0.99–16.70)	0.05
**Streptococcal toxic shock syndrome**					
No	12/195 (6.2)	1.00	0.04	1.00	
Yes	8/54 (14.8)	2.65 (1.02–6.87)		28.51 (3.21–253.14)	0.003
**ICU admission**					
No	7/126 (5.6)	1.00		1.00	
Yes	13/123 (10.6)	2.01 (0.71–5.86)	NS	5.75 (1.12–29.55)	0.04
**Received clindamycin**					
No	15/128 (11.7)	1.00		1.00	
0–24 hours post-admission	2/77 (2.6)	0.20 (0.04–0.90)	0.04	0.04 (0.003–0.55)	0.02
24–48 hours post-admission	2/30 (6.7)	0.54 (0.12–2.49)	NS	0.08 (0.007–1.07)	NS
>48 hours post-admission	1/14 (7.1)	0.58 (0.07–4.75)	NS	0.17 (0.01–2.53)	NS
**Surgical intervention**^c^					
None	18/153 (11.8)	1.00			
Major	2/57 (3.5)	0.27 (0.06–1.22)	NS		
Minor	0/39 (0)	0.00			
**Immunoglobulins**					
No	17/214 (7.9)	1.00	NS		
Yes	3/35 (8.6)	1.09 (0.30–3.92)			

CI: confidence interval; NS: not significant: ICU: intensive care unit.

^a^ for each additional year of age

^b^ for each additional point of Charlson score

^c^ Interventions defined as “major” were those considered as life-, limb- or organ-sparing (*e*.*g*. fasciotomy, drainage of empyema, hysterectomy), as opposed to ‘minor’ (*e*.*g*. joint lavage, abscess drainage for patients not meeting toxic shock criteria)

**Table 5:** Infection-related mortality and use of clindamycin among patients with group A *Streptococcus* infections according to site of infection.

Clinical diagnosis	Infection-related deaths/total	No clindamycin	Clindamycin 0–24 hrs post-admission	Clindamycin 24–48 hrs post- admission	Clindamycin >48 hrs post-admission
**Cellulitis**	5/51 (9.8%)	30 (59%)	13 (26%)	7 (14%)	1 (2%)
**Fasciitis/myositis**	2/35 (5.7%)	3 (9%)	30 (86%)	2 (6%)	0 (0%)
**Bone and joint**	0/24 (0%)	11 (46%)	6 (25%)	4 (17%)	3 (13%)
**Genital tract or GI**	2/35 (5.7%)	21 (60%)	9 (26%)	2 (6%)	3 (9%)
**Central nervous system**	1/4 (25.0%)	2 (50%)	2 (50%)	0 (0%)	0 (0%)
**Pneumonia/empyema**	9/62 (14.5%)	34 (55%)	13 (21%)	10 (16%)	5 (8.1)
**Endovascular**	0/12 (0%)	4 (33%)	3 (25%)	4 (33%)	1 8%)
**Primary bacteremia**	1/12 (8.3%)	12 (100%)	0 (0%)	0 (0%)	0 (0)
**Other**	0/14 (0%)	11 (79%)	1 (7%)	1 (7%)	1 (7%)

GI: gastro-intestinal

In multivariate analysis, increasing age, need for ICU admission and the presence of STSS were independently associated with death (Table 4), while Charlson score was confounded by age and no longer significant. Mortality tended to be higher in those with pneumonia/empyema. Mortality decreased over time, especially during the most recent period (2011–2016), and was lower in patients given clindamycin within 24 hours of admission. Albeit not reaching statistical significance, mortality was lower in those given clindamycin 24–72 hours after admission than amongst patients not given clindamycin. The protective effect of clindamycin was the same when analysis was restricted to patients admitted to the ICU (AOR: 0.04, 95%CI: 0.004–0.29, *P* = 0.002). When fitted into the model shown in Table 4, we could not demonstrate a beneficial effect of immunoglobulins (AOR: 1.66, 95%CI: 0.16–17.36, *P* = 0.67) or undergoing a surgical intervention (AOR: 0.21, 95%CI: 0.02–1.87, *P* = 0.16) on mortality. Subgroup analyses using our final model showed that the protective effect of clindamycin was similar in those with pneumonia/empyema (AOR: 0.06, 95%CI: 0.003–1.41, *P* = 0.08) compared to all others (AOR: 0.06, 95%CI: 0.007–0.62, *P* = 0.02), even if not significant in the former.

Table 6 shows similar analyses for patients with invasive GBS infection. Mortality increased with age and Charlson score but did not vary over time. In multivariate analysis, these two factors were independently associated with mortality (for each additional year of age, AOR 1.03, 95%CI: 1.01–1.06, *P* = 0.01; for each additional point in Charlson score, AOR 1.24 (95%CI: 1.06–1.45, *P* = 0.006). Here, we did not adjust for ICU admission, since we did not aim to measure the effect of clindamycin or intravenous immunoglobulins.

**Table 6:** In hospital infection-related mortality in patients with invasive group B streptococcal infections according to various characteristics.

	Deaths/Total (%)	Crude odds ratio (95% CI)	p-value
**Year of diagnosis**			
1996–2000	4/42 (9.5)	1.00	
2001–2005	3/66 (4.5)	0.45 (0.10–2.13)	NS
2006–2010	5/68 (7.4)	0.75 (0.19–2.98)	NS
2011–2016	12/128(9.4)	0.98 (0.30–3.23)	NS
**Age, years**			
		1.04 (1.02–1.07)^a^	0.003
0–17	0/53 (0.0)		
18–64	6/123 (4.9)		
65–79	9/77 (11.7)		
≥80	9/51 (17.6)		
**Sex**			
Female	14/141 (9.9)	1.00	
Male	10/163 (6.1)	0.59 (0.25–1.38)	NS
**Charlson score**			
0	1/99 (1.0)	1.33 (1.15–1.53)^b^	<0.001
1–2	3/83 (3.6)		
3–5	12/72 (16.7)		
≥6	8/50 (16.0)		
**Site of infection**			
All others	19/265 (7.2)	1.00	
Fasciitis/myositis	0/3 (0)	0.00	NS
Pneumonia/empyema	5/36 (13.9)	2.09 (0.73–5.99)	NS
**ICU admission**			
No	10/204 (4.9)	1.00	
Yes	14/100 (14.0)	3.16 (1.35–7.39)	0.01
**Received clindamycin**			
No	23/281 (8.2)	1.00	
Yes	1/23 (4.3)	0.51 (0.07–3.96)	NS
**Surgical intervention**^c^			
None	22/242 (9.1)	1.00	
Major	0/0 (0)	0.00	
Minor	2/62 (3.2)	0.33 (0.08–1.46)	NS
**Body mass index**		1.01 (0.96–1.06)^d^	NS

Confidence interval; NS: not significant; ICU: intensive care unit.

^a^ for each additional year of age

^b^ for each additional point of Charlson score

^c^ Interventions defined as “major” were those considered as life-, limb- or organ-sparing (*e*.*g*. fasciotomy, drainage of empyema, hysterectomy), as opposed to ‘minor’ (*e*.*g*. joint lavage, abscess drainage for patients not meeting toxic shock criteria)

^d^ for each additional point of body mass index

Table 7 displays risk factors for mortality among patients with invasive GCGS infections. Mortality increased with age and Charlson score. Although not statistically significant, mortality was lower in more recent periods compared to the years 1996–2000. In a multivariable analysis that adjusted for age (for each additional year of age, AOR 1.04, 95%CI: 1.00–1.08, *P* = 0.06), Charlson score was not significant and the only other independent correlate of mortality was the site of infection (compared to a baseline category including all other sites: for pneumonia/empyema, AOR 25.63, 95%CI: 2.45–268.34, *P* = 0.007; for fasciitis/myositis, AOR 4.20, 95%CI: 0.97–18.10, *P* = 0.054).

**Table 7:** In-hospital infection-related mortality in patients with invasive groups C or G streptococcal infections according to various characteristics.

	Deaths/Total (%)	Crude odds ratio (95% CI)	p-value
**Year of diagnosis**			
1996–2000	4/42 (9.5)	1.00	
2001–2005	3/66 (4.5)	0.45 (0.10–2.13)	NS
2006–2010	5/68 (7.4)	0.75 (0.19–2.98)	NS
2011–2016	12/128(9.4)	0.98 (0.30–3.23)	NS
**Age, years**			
		1.04 (1.02–1.07)^a^	0.003
0–17	0/53 (0.0)		
18–64	6/123 (4.9)		
65–79	9/77 (11.7)		
≥80	9/51 (17.6)		
**Sex**			
Female	14/141 (9.9)	1.00	
Male	10/163 (6.1)	0.59 (0.25–1.38)	NS
**Charlson score**			
0	1/99 (1.0)	1.33 (1.15–1.53)^b^	<0.001
1–2	3/83 (3.6)		
3–5	12/72 (16.7)		
≥6	8/50 (16.0)		
**Site of infection**			
All others	19/265 (7.2)	1.00	
Fasciitis/myositis	0/3 (0)	0.00	NS
Pneumonia/empyema	5/36 (13.9)	2.09 (0.73–5.99)	NS
**ICU admission**			
No	10/204 (4.9)	1.00	
Yes	14/100 (14.0)	3.16 (1.35–7.39)	0.01
**Received clindamycin**			
No	23/281 (8.2)	1.00	
Yes	1/23 (4.3)	0.51 (0.07–3.96)	NS
**Surgical intervention**^c^			
None	22/242 (9.1)	1.00	
Major	0/0 (0)	0.00	
Minor	2/62 (3.2)	0.33 (0.08–1.46)	NS
**Body mass index**		1.01 (0.96–1.06)^d^	NS

CI: Confidence interval; NS: not significant. GI: gastro-intestinal; GU: genito-urinary; ICU: intensive care unit.

^a^ for each additional year of age

^b^ for each additional point of Charlson score

^c^ Interventions defined as “major” were those considered as life-, limb- or organ-sparing (*e*.*g*. fasciotomy, drainage of empyema, hysterectomy), as opposed to ‘minor’ (*e*.*g*. joint lavage, abscess drainage for patients not meeting toxic shock criteria)

^d^ for each additional point of body mass index

## Discussion

This study demonstrates a significant burden of illness associated with beta-hemolytic streptococci. The average incidence rates of invasive GAS, GBS and GCGS infections were respectively 5.0, 6.0 and 4.3 per 100,000. In comparison, between 2001 and 2013 the incidence of invasive meningococcal infection in Quebec varied between 0.5 and 1.5 cases per 100,000 [1]. This study demonstrated a progressive rise in the incidence of iGBS and iGCGS, in contrast with iGAS infections that fluctuated without a discernible trend. This is consistent with reports from Europe and North America [2–6,18]. In keeping with previous literature [3,6], iGBS and iGCGS infections were associated with diabetes, being respectively 8.4 and 6.3 times more frequent in diabetics. GCGS infections occurred preferentially in older persons, especially males [2,4,6,19,20]. It seems possible that not only diabetes, but also immune senescence predisposes the elderly to iGCGS infections [21]. Changes in the incidence of iGBS and iGCGS infections were explained partially by an expansion of the high-risk population due to aging and increasing prevalence of diabetes, but not entirely as there was an age-adjusted rise among non-diabetics. Such data on disease burden could help define new target populations for GBS candidate vaccines initially designed to prevent neonatal disease [22] As there is currently no candidate vaccine against GCGS, our study also underlines the importance of determining whether future GAS and GBS candidate vaccines provide significant cross-protection against GCGS, since the latter are also associated with a significant burden of disease [22,23]. Although intrinsically less virulent than GAS, when causing invasive infections, the other β-hemolytic streptococci led to an identical infection-related mortality rate (8–9%) and similar durations of hospitalization, most likely because they infect older patients harbouring more co-morbidities and less physiological reserve.

This study provides useful insights into the treatment of invasive *Streptococcus pyogenes* infections with a large albeit retrospective cohort. It is recommended to add clindamycin based on animal models and in vitro data showing that it suppresses bacterial toxin production and cytokine release while remaining active even when bacterial growth has reached a stationary phase, in contrast with beta-lactams which then lose effectiveness [7,24]. However, evidence from clinical studies of a beneficial effect of clindamycin is scarce. Three small observational studies failed to show a statistically significant impact on mortality [8,25,26]. Among 62 patients admitted to the ICU, the lack of effect of clindamycin on mortality is mentioned in the abstract but actual numbers are not provided [26]. In a prospective population-based study with 77 patients, the AOR for the effect of clindamycin on mortality was not provided but was said to non-significant [25]. In the most recent one, a prospective population-based study with 84 cases, the AOR was of borderline significance (0.31, 95% CI: 0.09–1.12) [8]. Another small study (n = 67) showed a significant benefit in STSS [27]. In a study of 257 patients with iGAS infection, adding clindamycin reduced mortality, but only in patients with necrotizing fasciitis [28].

The current study suggests that indeed combining clindamycin to a beta-lactam reduces mortality in patients with invasive *Streptococcus pyogenes* infection. A potential bias is that there is less time to initiate clindamycin in patients with overwhelming infection, so that the rapid mortality could have prevented the use of clindamycin, rather than the other way around. We addressed this problem by stratifying cases according to the interval between admission and the first administered dose of clindamycin: its beneficial effect, albeit significant only in those treated within the first day, remained present when initiated later on. Our model adjusted for admission to ICU and anatomical site of infection, which took into consideration a potential confounding by indication. In patients with proven or suspected iGAS infection, clindamycin as an adjunctive treatment is more commonly used in sicker patients, and indeed our patients admitted to ICU were over twice more likely to have received this drug than others. Adjustment for age and year of diagnosis should have also controlled for other putative confounders by indication, including variation of clinician’s practices over time or according to the patient’s age with regards to therapeutic conduct .

Roughly half of the patients did not receive adjunctive clindamycin, including three fourths of those who died from GAS infection. It seems possible that a more frequent use of this adjuvant treatment at an early stage in patients with community-acquired septic shock of unknown etiology and/or severe pneumonia requiring ICU management might prove life-saving for some. In such cases, the time-sensitive nature of this benefit might justify its administration before the streptococcal etiology becomes known. For instance, while 86% of patients with necrotizing fasciitis/myositis received clindamycin within 24 hours of admission, only 22% of their counterparts with pneumonia/empyema benefited from this simple intervention. The higher mortality in patients with pneumonia, even after adjusting for age and clindamycin use, may also be related to the impossibility of surgically removing necrotic tissues, in contrast with cases of necrotizing fasciitis/myositis.

As others before, we could not demonstrate any beneficial effect of polyclonal immunoglobulins on survival among patients with invasive GAS infection, but our power to do so was limited since only 35 patients received this treatment. An early study supporting immunoglobulins was criticized for using historical controls with an unusually high mortality (66%) [29]. Unfortunately, a randomised trial was terminated early due to low enrollment [30]. A large (n = 192) retrospective study in children with STSS did not show any benefit of immunoglobulins, but had a small number of deaths [31]. Of the four small studies mentioned previously, three failed to show a benefit of immunoglobulins while one did [8,25–27]. A recent study using administrative databases showed no benefit of immunoglobulins in patients with necrotizing fasciitis [32]. Given the lack of supporting evidence, this expensive treatment was abandoned progressively in our region. Nevertheless, the case-fatality rate of iGAS infection dropped, especially during the last five years, presumably due to better awareness of this condition among physicians, more systematic ICU admission and improvement in critical care life-support measures. Perhaps reflecting improving management over the years, patients from this study with necrotizing fasciitis or STSS had a case-fatality rate that was lower than in some other studies (STSS: 26%-44%; necrotizing fasciitis: 10%-34%). This was not the case for patients with pneumonia (15%-38%) [19,21,31,33–34].

The main limitation of our study was its retrospective nature, which could have led to imprecision in assessing some variables, such as Charlson score, because of missing values. The inclusion of genital culture specimens, and thus isolates from nonsterile sites, could represent another limitation. Given the very low rate of genital colonization with GAS (only 0.03% to 0.06%) and the well-recognized nature of puerperal sepsis as an invasive form of streptococcal disease, we would argue that this is justified, as the isolation of GAS is unlikely to be due to chance alone in a female patient with a compatible clinical presentation [35–36]. For GCS and GGS, colonization rates may be higher (1–3%), but out of 9 cases with GCGS endometritis, 5 had a positive blood culture and exclusion of the remaining 4 would not impact our results, as the treatment effect of clindamycin was limited to the subset of patients with iGAS disease. We chose to use in-hospital infection-related mortality as the main outcome. While this endpoint is in part subjective, we felt this was preferable to using all-cause 30-day mortality. The latter would have included a few cases whose deaths were caused by unrelated processes (brain death, metastatic cancer), which could have prompted clinicians not to treat iGAS. Other limitations include the absence of species-specific or molecular identification for GCGS isolates. Unlike iGAS, which is a provincially reportable disease, these organisms are not routinely forwarded to a reference laboratory. While this could have resulted in cases due to species other than *S*. *dysgalactiae* ssp. *equisimilis* being included, these species rarely cause human disease and are unlikely to have significantly influenced the incidence rates.

## Conclusion

In conclusion, the current study, which examined invasive beta-hemolytic streptococcal infections in a well-defined population over two decades, documented an increasing incidence of iGBS and iGCGS infections, partially related to an increasing prevalence of diabetes in the elderly. No such secular change was seen for invasive GAS infections. Among patients with invasive GAS infections, the in-hospital mortality decreased over time, presumably because of better case-management. In patients with invasive *Streptococcus pyogenes* infection, the addition of adjunctive clindamycin to a beta-lactam decreased mortality, while immunoglobulins seemed ineffective. The highest mortality was seen in patients with GAS pneumonia/empyema, for whom clindamycin was also protective but underused.
